# Assessing Patterns and Risk to Chilean Freshwater Fish Distributions Using Multi‐Species Occupancy Models

**DOI:** 10.1002/ece3.71719

**Published:** 2025-07-15

**Authors:** Erin E. Tracy, Evelyn Habit, Konrad Górski, Nann A. Fangue, Andrew L. Rypel

**Affiliations:** ^1^ Department of Wildlife, Fish & Conservation Biology University of California Davis Davis California USA; ^2^ Departamento de Sistemas Acuáticos Facultad de Ciencias Ambientales, Universidad de Concepción Concepción Chile; ^3^ Centro de Ciencias Ambientales EULA‐Chile Universidad de Concepción Concepción Chile; ^4^ Instituto de Ciencias Marinas y Limnológicas Facultad de Ciencias, Universidad Austral de Chile Valdivia Chile; ^5^ Facultad de Ciencias, Universidad Católica de la Santísima Concepción Concepción Chile

**Keywords:** aquatic conservation, biodiversity, data‐limited, freshwater fish, multispecies occupancy models, rivers

## Abstract

To advance our understanding of freshwater biodiversity in data‐limited systems, this study used multispecies occupancy models to predict species richness and individual species occupancy, providing critical insights for the conservation of these rapidly declining ecosystems. Chilean watersheds were chosen as the study system as they support a highly endemic and vulnerable assemblage of freshwater fishes in need of increased research and management. We tested several model types and ultimately pursued latent spatial multispecies occupancy models, which gained popularity in wildlife ecology, but are relatively underutilized in fisheries ecology. Advantages include simultaneously modeling multiple species to infer both species‐specific and assemblage‐level responses to hydro‐geomorphological conditions while also accounting for imperfect species detections. Model results showed that fish species richness is primarily driven by negative relationships with elevation; however, individual species responses were variable across all environmental drivers. We present maps of predicted occupancies, representing niche model results for selected native and nonnative species. Finally, to put our results in the context of the rapid development in hydropower taking place throughout Chile, we analyzed predicted species richness and occupancy patterns in relation to aquatic ecosystem fragmentation risk from current and planned dams throughout Chile. Results showed a large number of dams are planned for the diverse lower elevation areas of the Biobio, Valdivia, and Puelo River Basins, highlighting the potential for negative impacts to the species that inhabit them. As over half the species modeled are currently listed as endangered, critically endangered, or data deficient by the International Union for Conservation of Nature (IUCN), model outputs could aid in conservation planning. This approach not only enhances our ability to protect Chile's unique and vulnerable freshwater fish species but also provides a robust framework for integrating modeled ecological insights of data‐limited systems into conservation planning.

## Graphical abstract

To advance our understanding of freshwater biodiversity in data‐limited systems, this study used multispecies occupancy models to predict species richness and individual species occupancy, providing critical insights for the conservation of these rapidly declining ecosystems. By synthesizing limited fisheries sampling data and hydro‐geomorphological conditions, results of this study lead to a broader understanding of freshwater fish biodiversity in Chile and can inform aquatic ecosystem conservation and management. This approach not only enhances our ability to protect Chile's unique and vulnerable freshwater fish species but also provides a robust framework for integrating modeled ecological insights of data‐limited systems into conservation planning.

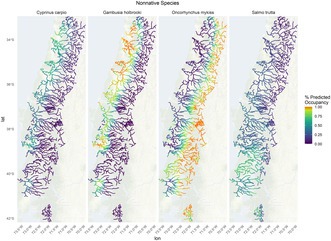

## 1 Introduction

Riverine stream networks are social‐ecological systems that support diverse flora and fauna communities and provide valuable ecosystem services (Parsons et al. 2016; Pascual et al. 2017; Perry et al. 2024). Unfortunately, climate change coupled with other anthropogenic disturbances that affect rivers and their surrounding catchments has resulted in rivers being classified as one of the most threatened ecosystems on Earth (Dynesius and Nilsson 1994; Palmer et al. 2009; Barbarossa et al. 2020; Intergovernmental Panel On Climate Change (IPCC) 2023). This trend is particularly concerning given that freshwater habitats support one third of all described vertebrate species that have declined at more than twice the rate of other vertebrates (Grooten and Almond 2018; Albert et al. 2021). These species now make up almost a third of the species listed as at risk of extinction on the IUCN Red List (IUCN 2023). Addressing this biodiversity crisis requires innovative tools to assess species distributions and biodiversity patterns, particularly in regions where environmental regulations and resources for conservation are limited (Winemiller et al. 2016; Colin et al. 2022; Habit et al. 2022).

In data‐limited regions like Chile, where river ecosystems face increasing anthropogenic pressure (e.g., hydropower, urbanization, introduced species, climate change), there is an urgent need for tools that can translate patterns of freshwater biodiversity into actionable management strategies (Linke et al. 2019; Harper et al. 2021; Cooke et al. 2022). Chile has a relatively species‐poor yet highly endemic (81%) assemblage of 43 native freshwater fish species due to long‐term geographic isolation (Atacama Desert to the north, Andes to the east, and Pacific Ocean to the west and south) (Dyer 2000; Habit et al. 2007; Vargas et al. 2015). Most of these native fish are small‐bodied with specific habitat and feeding requirements and limited distribution ranges, which make them particularly vulnerable to the impacts of disturbances (Habit et al. 2010, 2019, 2024; Vila and Habit 2015). Compounding these disturbances is the lag in environmental legislation for river conservation, which has not kept pace with the increasing hydropower developments or changing climate (Bauer 2009). Developing and applying tools to assess riverine biodiversity and resilience in this context is critical to maintaining river ecosystem functions and services under future watershed disturbances (Grantham et al. 2019; Perry et al. 2024).

To address these challenges, innovative modeling approaches are needed to better understand biodiversity patterns and species distributions while accounting for the complex environmental and ecological factors shaping freshwater ecosystems. Multispecies occupancy models (MSOMs) are widely used in wildlife ecology to predict species distribution and understand species–habitat relationships (Doser et al. 2023). MSOMs are regression‐based models that leverage the pervasive and important impact of environmental filters (e.g., hydro‐geomorphic variables driving biodiversity patterns) on species distribution predictions (Tonn and Magnuson 1982; Poff 1997; Rypel 2023). The models jointly predict multiple species occurrences along with species detection probabilities as a function of habitat variables and detections of other species (MacKenzie et al. 2017). The process of modeling multiple species within a single model allows for residual species correlations which ultimately increases the precision of individual species distributions and community metrics. Additionally, MSOMs explicitly accounts for imperfect detection separately from true species occurrence and uses detection‐nondetection data from multiple species as random effects arising from community‐level distributions (Gelfand et al. 2005). Finally, these models account for spatial autocorrelation by including structured random effects, which are often necessary when modeling species distribution across a large spatial extent (Latimer et al. 2009).

MSOM is primarily deployed to directly consider the influence of habitat, disturbance and land‐use change, and environmental gradients on metacommunity structure (Zipkin et al. 2010; Mattsson et al. 2013; Mihaljevic et al. 2015; MacKenzie et al. 2017). However, they show promise to address similar ecological questions and influence management actions in the fisheries field. MSOMs could be especially useful in areas where sampling data are limited (e.g., infrequent surveys, limited fish life history data) (Wagner et al. 2020). There are only a few examples of marine (Holt et al. 2013; Coggins Jr. et al. 2014) and freshwater (Potoka et al. 2016; White et al. 2020; Wagner et al. 2020; Wohner et al. 2023) multispecies distribution models. While generalized linear models have frequently been used to analyze relationships between habitat and individual fish occurrence, they do not account for species interactions and can lead to overly simplistic estimations of species distribution (Latimer et al. 2009; Wagner et al. 2020). As a result, multi‐species models tend to better predict species presence or absence compared with models that do not account for species interactions and can therefore be used to gain deeper insight into ecological community dynamics (Wagner et al. 2020). Another advantage is that detection and occupancy probabilities for rare species are explicitly quantified (Broms et al. 2016; White et al. 2020).

By leveraging MSOMs, our ultimate goal is to advance river and fish conservation by improving our understanding of species distributions and the environmental factors that influence them. This study aims to achieve this goal by using MSOMs to explore how hydrogeomorphological settings affect fish assemblage species diversity, providing a pathway to better understand species distribution patterns. The study region encompasses Andean rivers in Chile between 32° and 42° S, as the rivers in this area offer a unique geomorphological setting and have been isolated for more than 10,000 years. This research (1) creates predictive maps for native and nonnative species richness, (2) generates predictive occupancy maps for individual species of special conservation concern, and (3) overlays anthropogenic disturbances such as dams on predicted species richness and individual species occurrence maps to support conservation decision making. Results from these analyses can be used to guide researchers and managers in setting priorities for protecting or restoring freshwater ecosystems and native fishes.

## 2 Methods

### 2.1 Study Region

The study region encompasses Andean rivers in Chile between 32° and 41° S in latitude. This region includes 11 large river drainage basins (Maipo to Puelo) that vary in underlying geomorphology, flow regime, and level of disturbance. The climate is predominately a warm‐summer Mediterranean climate (Beck et al. 2018), which can vary due to climatic oscillations (e.g., El‐Niño, Pacific Decadal, Antarctic Oscillations) and is strongly influenced by topography, including the Andes Cordillera, Central Valley, and Coastal Cordillera. The study region contains over half of the total human population of Chile as well as the majority of cultivated land (72% of farms, 54% of forest plantations) (Bonilla and Johnson 2012). Human impacts in the areas cause a variety of ecological disturbances. For example, during the primary irrigation season, water extractions can limit river flow (Habit et al. 2022). Additionally, while the majority of rivers in this study are free flowing, the study area contains over half (91 of 148) of the hydropower plants in Chile, which are primarily located on the Rapel, Maule, and Biobío river drainage basins.

### 2.2 Data Collection

The initial data set used in this analysis included fish sampled from 2015 through 2023 using a variety of standard methods (backpack electrofishing, seines, fykes, and gill nets) with sampling efforts unequally distributed across the 11 river drainage basins. After collection, fish specimens were kept in containers filled with cold, oxygenated water while in the field. All specimens were sedated using BZ‐20 (ethyl‐p‐aminobenzoate), then measured for total length and weighed at the collection site. Following this, they were placed back in clean water containers. Once they fully recovered, all specimens were returned to their natural habitats (Orrell et al. 2025). Euthanasia was performed only when necessary, such as if a fish did not recover satisfactorily after sedation. In such cases, an overdose of the same anesthetic was used for euthanasia. Fish data were then subset to include only electrofishing data collected during the low flow period (Dec–April) of 2021 to meet the closed population assumption of an MSOM. The year 2021 was chosen as it had the most repeat samples (a little over a third of all sites were sampled more than once). 10,428 fish individuals representing 25 species (19 native, 6 nonnative) were collected in 122 sampling sites in 9 river drainage basins (Maipo, Rapel, Mataquito, Maule, Itata, Biobío, Imperial, Toltén, and Valdivia) (Table 1).

**TABLE 1:** List of species included in MSOM model with status, total detections, and IUCN conservation status.

Scientific name	Status	Total detections	IUCN status
*Percilia irwini*	Native	1882	Endangered
*Percilia gillissi*	Native	1430	Endangered
*Diplomystes arratiae*	Native	61	Endangered
*Diplomystes incognitus*	Native	1	Endangered
*Cheirodon kiliani*	Native	1	Endangered
*Cheirodon australe*	Native	476	Vulnerable
*Diplomystes camposensis*	Native	237	Vulnerable
*Basilichthys microlepidotus*	Native	234	Vulnerable
*Aplochiton marinus*	Native	11	Vulnerable
*Percichthys trucha*	Native	663	Least Concern
*Galaxias maculatus*	Native	468	Least Concern
*Bullockia maldonadoi*	Native	273	Least Concern
*Cheirodon galusdae*	Native	198	Least Concern
*Galaxias platei*	Native	50	Least Concern
*Hatcheria macraei*	Native	5	Least Concern
*Brachygalaxias bullocki*	Native	2	Least Concern
*Aplochiton taeniatus*	Native	1	Least Concern
*Trichomycterus areolatus*	Native	2102	Data Deficient
*Geotria australis*	Native	324	Data Deficient
*Oncorhynchus mykiss*	Nonnative	998	Nonnative
*Gambusia holbrooki*	Nonnative	579	Nonnative
*Cnesterodon decemmaculatus*	Nonnative	189	Nonnative
*Salmo trutta*	Nonnative	161	Nonnative
*Australoheros facetus*	Nonnative	50	Nonnative
*Cyprinus carpio*	Nonnative	32	Nonnative

A suite of abiotic data at the catchment (e.g., elevation, geology, rainfall), valley (e.g., valley and trough width and slope), and channel scales (e.g., belt width, sinuosity, wavelength) were also collated and analyzed from existing data sets using software tools. We used ArcGIS tools to extract geomorphic data from our study system at the catchment, valley, and channel scales at 2–5 km intervals (Harris et al. 2009). Catchment scale variables including elevation were estimated from a 12.5 m digital elevation model (DEM) from the Natural Resources Information Centre of Chile, geology measured from 1:1.000.000 scale vector geology map aggregated into five sediment types, and mean annual precipitation from WorldClim (https://www.worldclim.org/data/index.html). We calculated valley and channel scale variables from transects using the Digital Shoreline Analysis System (Habit et al. 2022). Finally, the current and future barrier distribution was obtained from the Chilean Ministry of Energy based on hydropower potential and water rights with current dams as of 2018 and planned dams by 2050.

### 2.3 Analysis

#### 2.3.1 Multispecies Occupancy Models

Given the large spatial distribution of the data in this study it was imperative to explicitly account for spatial autocorrelation by including spatially structured random effects in the model (Banerjee et al. 2003; Shirota et al. 2019; Doser et al. 2023). Additionally, because sampling methodology was not always consistent between surveys and the spatial distribution of sampling was uneven, species presence absence data were used instead of abundance and imperfect detection was accounted for in the model (Doser et al. 2023). For example, while the data were subset to only include fish collected with the same standard method during the low flow period of 2021 some regions of the study area were sampled more heavily than others due to different accessibility or resource constraints introducing potential bias. Studies show that accounting for residual species correlations, imperfect detection, and spatial autocorrelation when present in the data and model leads to better predictive performance and therefore the spatial factor MSOM was tested along with several others including an MSOM and a latent factor MSOM (Doser et al. 2022, 2023).

To find the most parsimonious model with the best predictive performance across the region, we followed a model selection procedure to identify the most influential covariates. To do this, we calculated a matrix of Pearson product moment correlations using an initial set of 12 geomorphological variables at multiple spatial scales (valley, channel). Results indicated several variables were significantly correlated with one another; thus each correlated variable was separately run through the models to assess its Widely Applicable Information Criterion (WAIC) using the spOccupancy R package and the correlated variable with the best score was retained (Doser et al. 2022). Variables with the lowest WAIC were then combined in a model, and several iterations of that model tested until the best WAIC score was found. This technique followed prior research (Hooten and Hobbs 2015; Stewart et al. 2023) indicating information criteria such as WAIC values can be informative toward selecting occupancy covariates if the purpose of the model is prediction. Finally, before integrating covariates into the model, they were scaled (mean of 0 and standard deviation of 1) using the R scale function.

#### 2.3.2 Prediction and Mapping

We used the predict function in the spOccupancy R package to generate a series of posterior predictive samples at new locations, given a set of covariates and spatial locations. When predicting new values using a latent factor, MSOM predictions were made at both sampled and nonsampled locations using latent factors. Using this function, predicted values of detection probability for each species given their relationship to the covariates were generated (Doser et al. 2022). Then maps of species richness predictions for native species and all species, as well as individual species predicted occupancy maps, were made. Endangered, data deficient, and nonnative species were selected for the creation of individual species occupancy maps from species that had sufficient (> 10) detections (Table 1). Finally, the native species richness map was then used as the base layer with a current and planned dams layer overlayed to descriptively assess potential impacts.

## 3 Results

### 3.1 Model Fit and Covariates

The spatial latent multispecies occupancy model had the lowest WAIC by accounting for residual species correlations, imperfect detection, and spatial autocorrelation (Watanabe 2010). Model results showed that values were < 1.1, and the effective sample size (ESS) values were sufficiently large. Trace plots of individual model parameters were normal, and posterior summaries indicated that one latent factor loading was the best fit for this data set. Latent factors are unmeasured site‐specific covariates that are treated as random variables in the model estimation procedure. They account for unmeasured environmental and behavioral interactions in predicted species occurrence. Posterior predictive checks were used to assess the model's goodness of fit and ensure the model produced data closely aligned with observed data (Hooten and Hobbs 2015). Our community‐level Bayesian probability value was 0.42 indicating adequate model fit.

**FIGURE 1:**
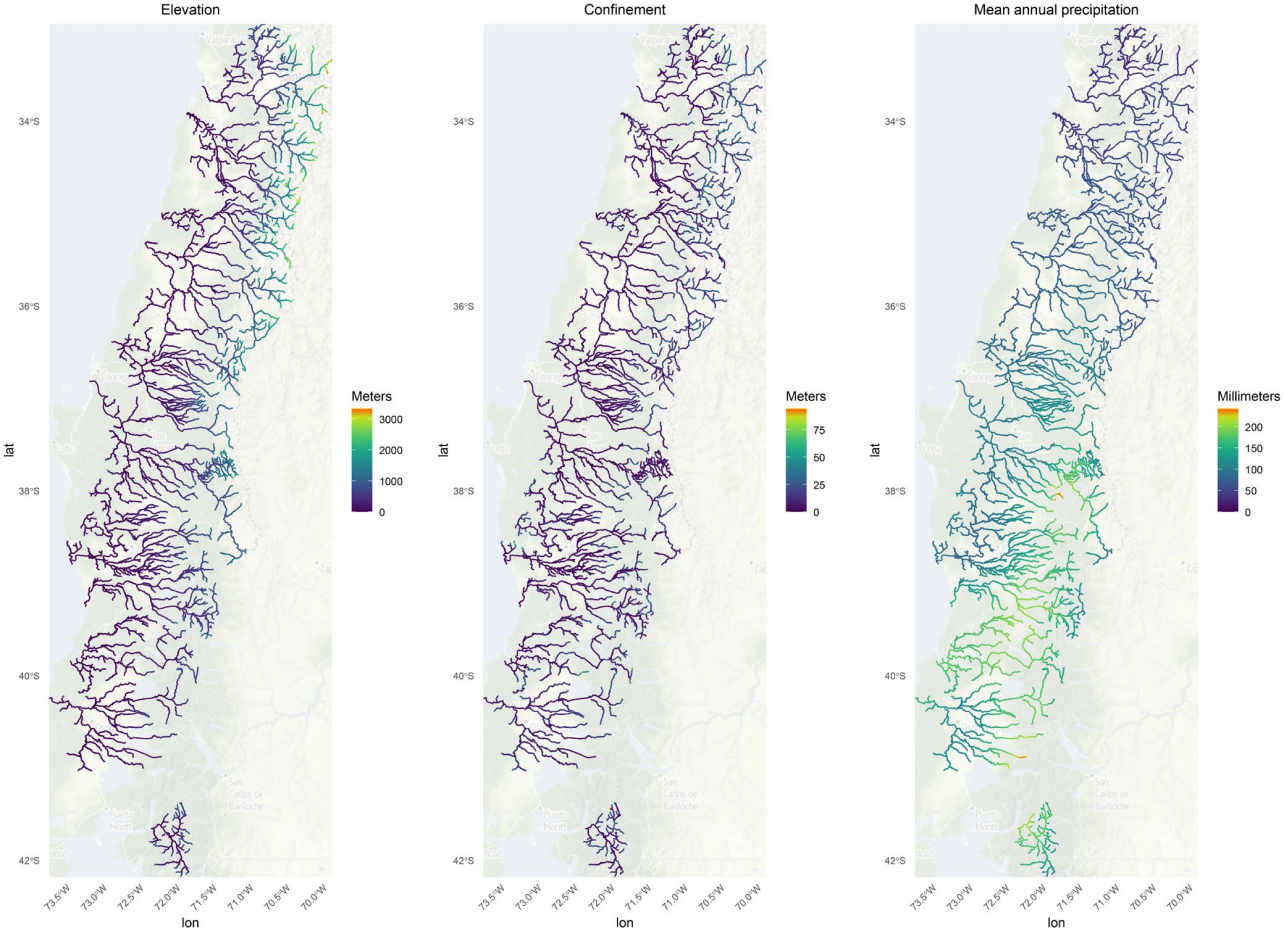
Covariate maps of elevation, confinement, and mean annual precipitation. Maps exclude the top 2% of outliers to better visualize their range. Warmer colors indicate higher values.

The best fitting model included three covariates (elevation, mean annual precipitation, and confinement, that is, ratio of valley width to valley floor width) (Figure 1). Maps of variation in covariate value range are displayed in Figure 1. Exploring the community‐level model output, the variance in the occurrence intercept term is high (mean = 12.47) indicating considerable variability in species occurrence across the study area. Elevation (mean = −1.44) had a significant negative relationship with species richness, with moderate variability (mean = 3.13) in how it affects species occurrence across sites. Mean annual precipitation was not significant but had a weak negative relationship with species richness (mean = −0.10), with a somewhat high variability in its influence on species occurrence (mean = 6.08), suggesting its impacts are context specific. Finally, confinement had a not significant very weak positive effect (mean = 0.04), with low variability (mean = 0.20) indicating that its weak relationship with occurrence was relatively consistent across species (Tables S1 and S2). While we also had access to hydrologic data of interest (flow and hydropeaking data), these variables were at a far coarser scale than the geomorphological variables, and model results indicated they did not significantly impact fish occurrence. Model results for individual species highlighted the value of accounting for imperfect detection, with estimated detection probabilities ranging from 20% to 80%, depending on the species (Table S2).

### 3.2 Species Richness

**FIGURE 2:**
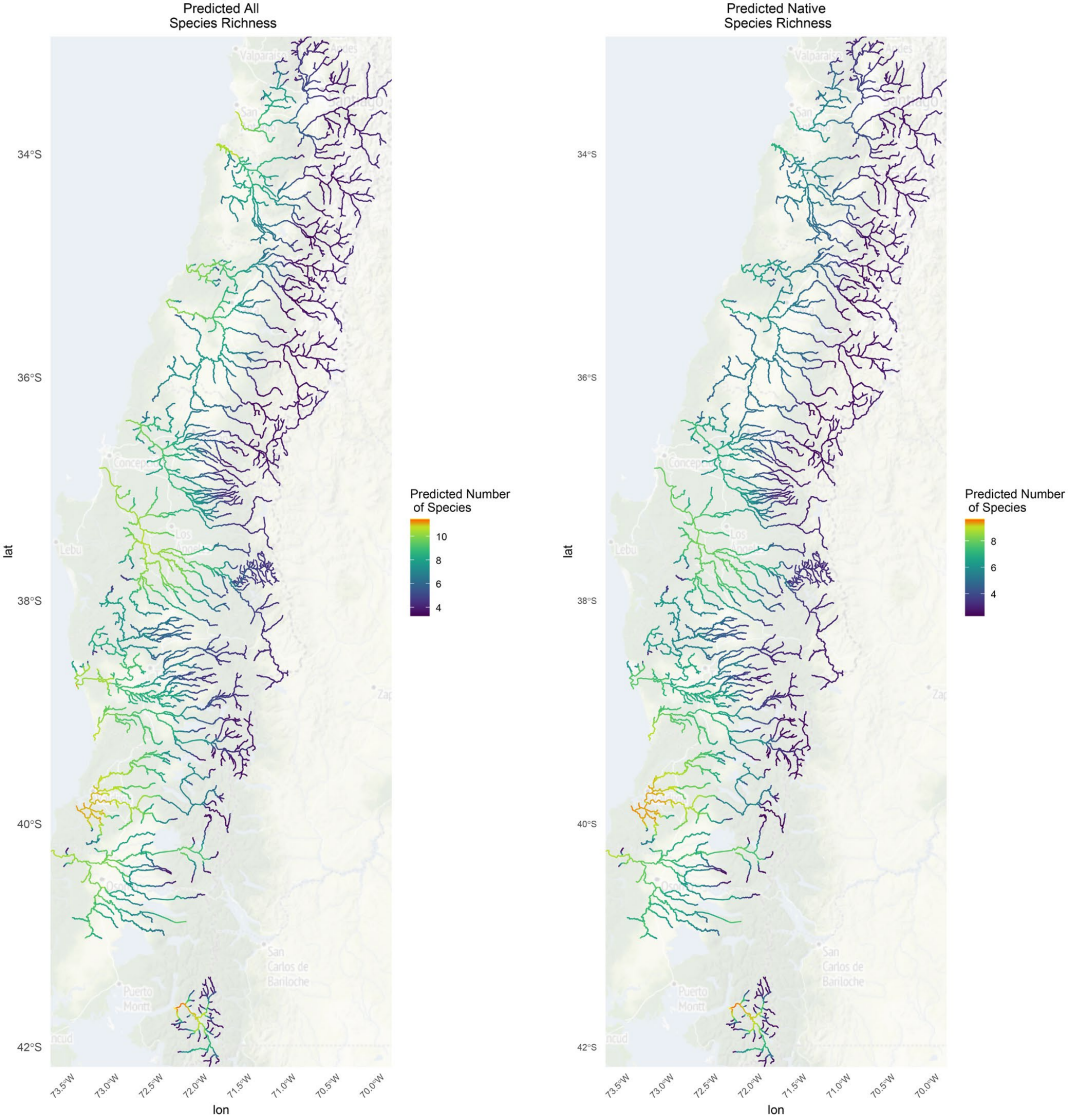
Multispecies occupancy model predictions of total species richness (left) and native only richness (right) of Chilean freshwater fishes. Warmer colors (orange) represent high‐predicted species richness.

Elevation had a significant negative effect on species richness, with the most species assemblages occurring at low elevations (> 1000 m). While not statistically significant in the model, mean annual precipitation did have a weak negative relationship with species richness at the community level (Table S1, Figure 1). Predicted native species richness follows parallel trends to that of all species richness, with a slightly lower maximum predicted number of species (a maximum of 9.6 native species compared to a maximum of 11.4 total predicted species) (Figure 2).

### 3.3 Individual Species Occupancy Maps

#### 3.3.1 Nonnative Species

**FIGURE 3:**
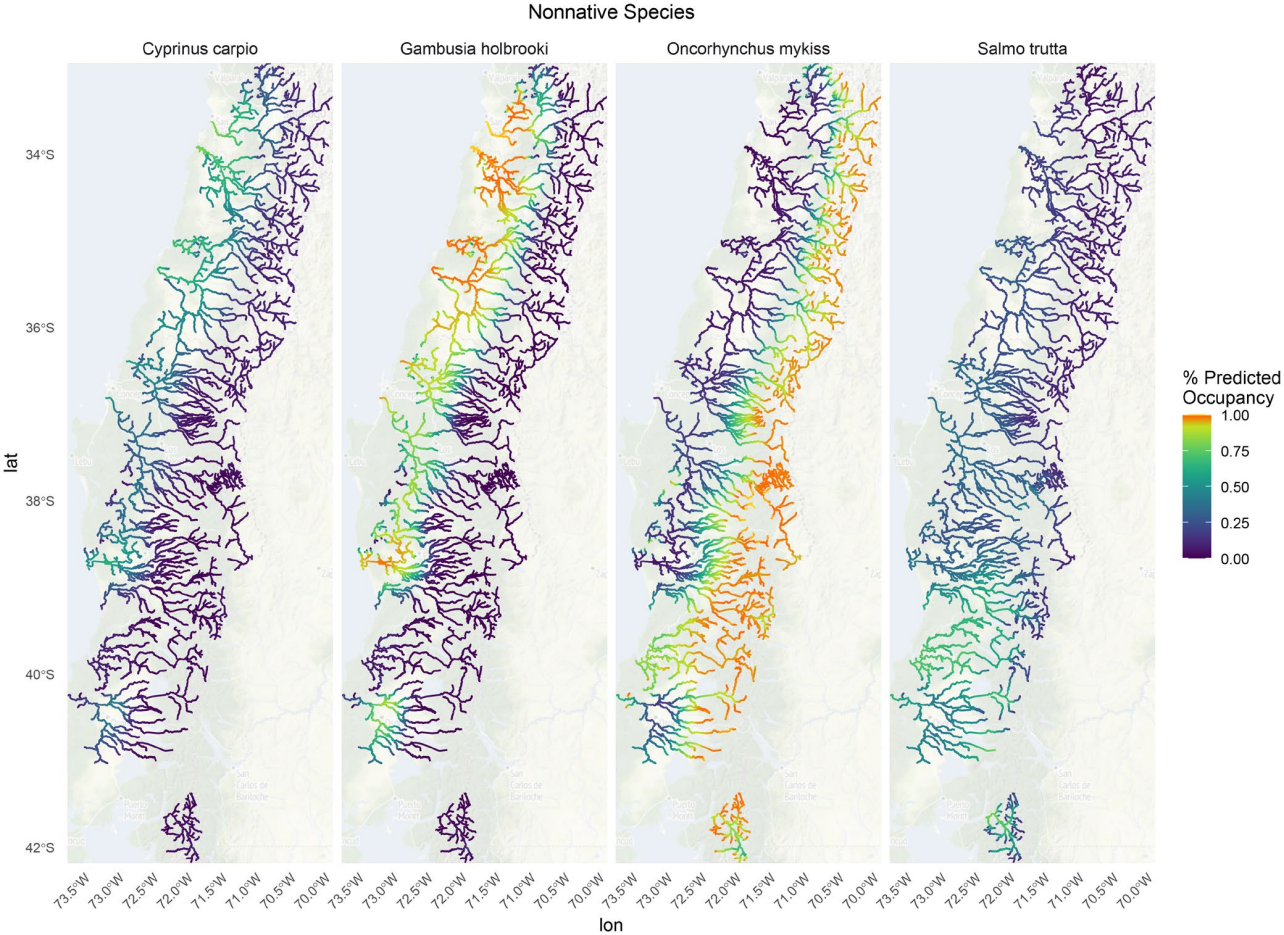
Multispecies occupancy model predictions of nonnative species occurrence including *Cyprinus carpio* (left), *Gambusia holbrooki* (inner left), *Oncorhynchus mykiss* (inner right), and *Salmo trutta* (right). Warmer colors (orange) represent high‐predicted occupancy.

*Cyprinus carpio* had a relatively low detection probability (mean 45%) with a large confidence interval (13%–88%) (Figure 3). *Gambusia holbrooki* (mean 82% detection probability) had a significant negative relationship with elevation and mean annual precipitation and is therefore predicted to occur at higher rates in lower elevations and in more northern regions with lower mean annual precipitation (Figure 3) with slightly high‐predicted occurrences in lower elevation rivers. Salmonid species, including *S. trutta* (mean 68%) and *O. mykiss* (mean 90%) had relatively high detection probabilities. *Oncorhynchus mykiss* also had significant positive relationships with elevation and mean annual precipitation.

#### 3.3.2 Data Deficient Species

**FIGURE 4:**
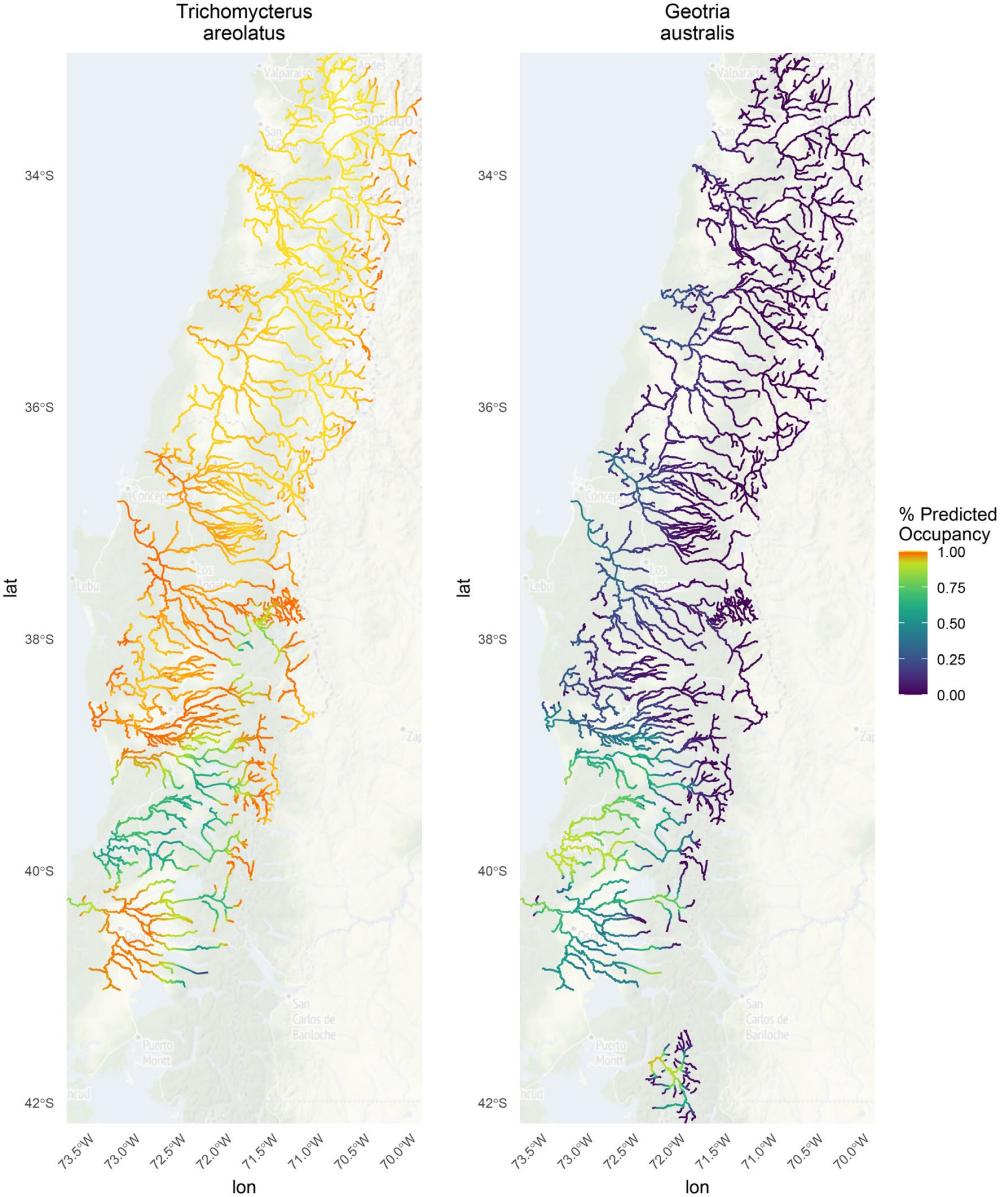
Multispecies occupancy model predictions of data deficient species *Trichomycterus areolatus* (left) and *Geotria australis* (right). Warmer colors (orange) represent high‐predicted occupancy.

*Trichomycterus areolatus* had a significant negative relationship with precipitation, with high‐predicted probabilities of occurrence throughout the study area, likely because these species occurred frequently in the data set used in the model (2102 individuals found across 9 basins; Figure 4). *Geotria australis* occurrence had a significant negative relationship with elevation and a significant positive relationship with mean annual precipitation, a pattern that is readily observable in the southernmost part of the study area (Figure 4).

#### 3.3.3 IUCN Endangered Species

**FIGURE 5:**
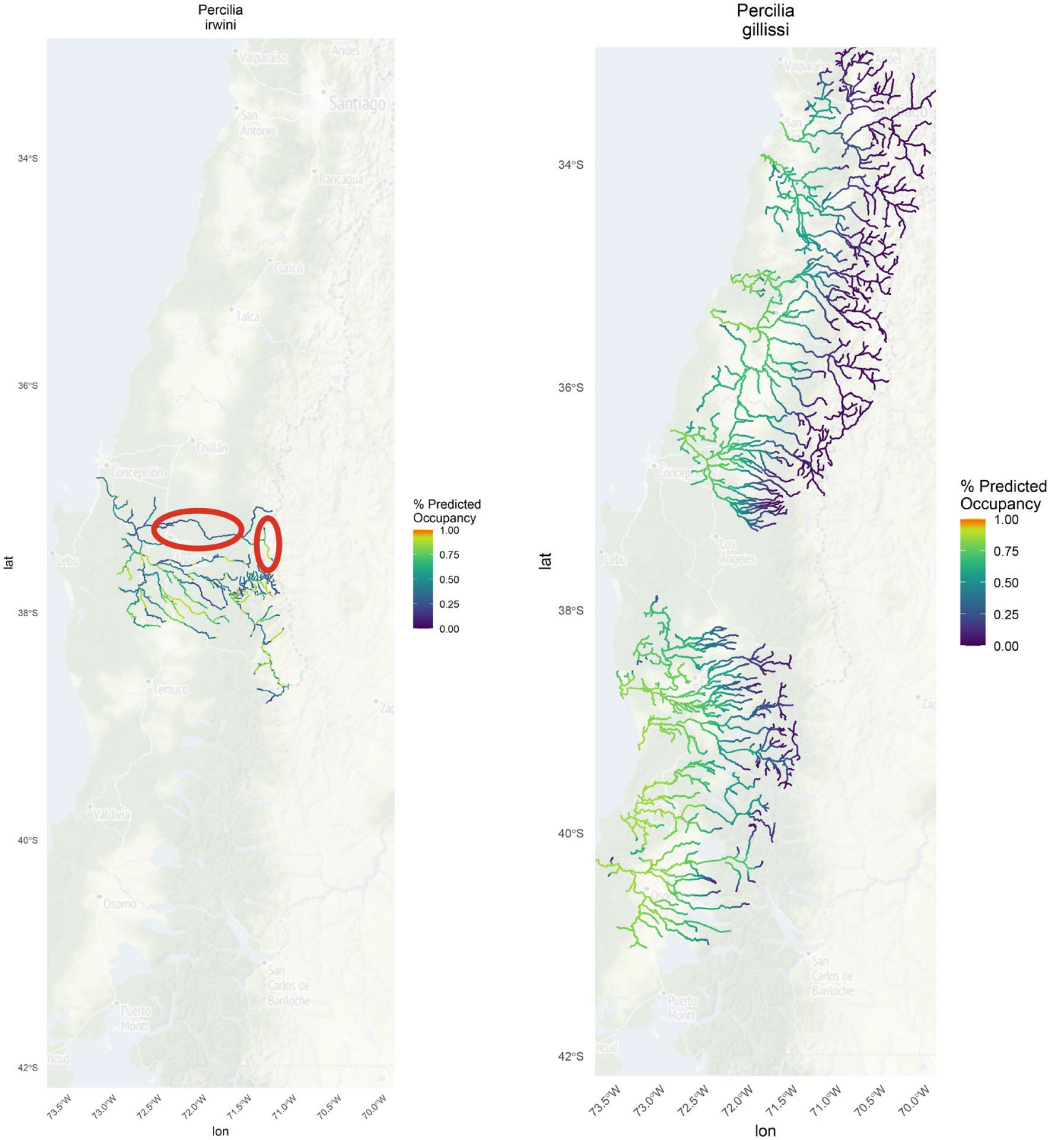
Multispecies occupancy model predictions of species classified by IUCN as “endangered” *Percilia irwini* (left) and *Percilia gillissi* (right). Warmer colors (orange) represent high‐predicted occupancy.

The endangered *P. gillissi* ranges from the Aconcagua River drainage basin (32° Latitude South) to the Maullín River drainage basin (41° Latitude South) with the exception of the Biobío River (Dyer 2000), whereas the endangered *P. irwini* has a more limited geographic range, primarily inhabiting the Biobío River drainage basin (Habit et al. 2007). In our model, *P. gillissi* had high detection probabilities (mean 95%) and a significant negative relationship with elevation, resulting in higher rates of predicted occupancy at lower elevations along the coast (Figure 5). *Percilia irwini* also had a high detection probability (mean 97%) and high‐predicted occupancy across its small range (Figure 5). However, problem areas with the *P. irwini* model predictions were identified by expert opinion (circled in red) including high‐predicted occurrence in Laja Lake where the species is known to be absent and low‐predicted occurrence in the Laja River sub‐basin where the species is known to be abundant (Figure 5).

### 3.4 Dam Impacts

**FIGURE 6:**
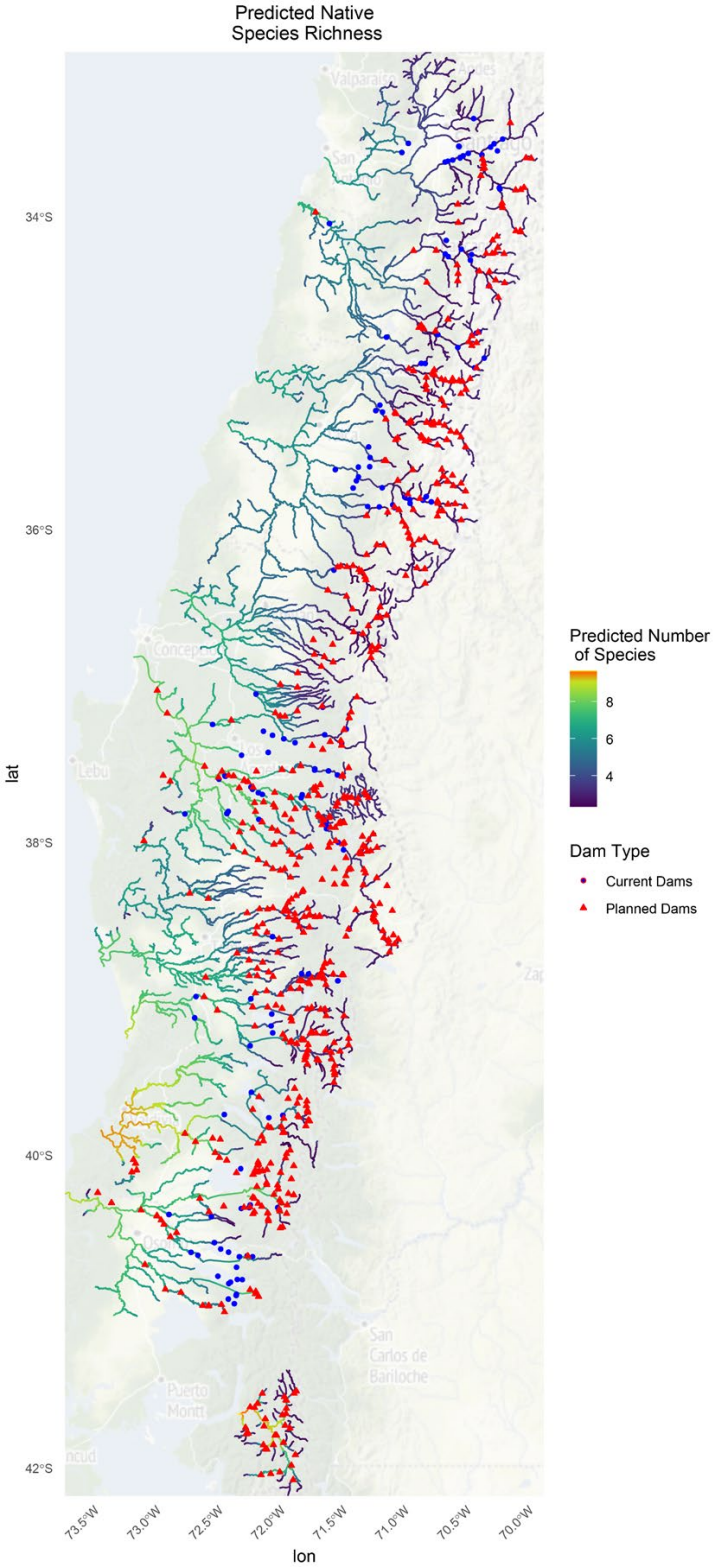
Multispecies occupancy model predictions of total species richness overlayed on current and planned dams in Chile. Warmer colors (orange) represent high‐predicted occupancy, red triangles are potential dams, and blue circles are current dams.

The location of current and planned dams in Chile (Figure 6). There are currently four times more planned dams (638 planned, 147 current) as of the 2018 Chilean Ministry of Energy data set. New dams are planned for all 11 basins in the study area impacting all the fish they support, including the endangered species *P. irwini* found only in the Biobío River Drainage Basin. Many of the planned dams appear to be in the higher elevation reaches of the rivers where species richness is lower. However, there are a large number of dams planned for the highly diverse lower elevation areas of the Biobío, Valdivia, and Puelo River Drainage Basins that will experience the highest impacts to biodiversity.

## 4 Discussion

To better understand and protect our rapidly disappearing freshwater biodiversity, this study used multi‐species occupancy models to predict species richness and individual species occupancy in a data‐limited study area. Because the loss of native freshwater fish diversity is especially acute in countries like Chile, country‐specific data on its unique endemic species are needed to guide conservation. Results of this study indicated that species richness was primarily driven by elevation and predicted occupancies for select native and nonnative species to evaluate their distribution in the context of conservation planning, including threats from hydropower. By leveraging these models, our study provides critical insights into the drivers of fish assemblage diversity in Chilean rivers, contributing valuable information for mitigating the impacts of ongoing environmental change and supporting more strategic, conservation‐focused river management.

### 4.1 Species Richness

Species richness is an important component of freshwater ecosystem resilience and can be used to guide conservation efforts in the face of disturbance and global change (Kerr et al. 2007, 2010; Myers et al. 2021). Given the lack of widespread freshwater fish monitoring and assessment in many watersheds and the difficulty of detecting all present species in a given survey, multi‐species occupancy models can be a useful tool for predicting species richness across riverscapes. One strength of these models is the ability to capture complex species–environment relationships through the integration of environmental covariates previously shown to drive fish diversity patterns (Oberdorff et al. 1995). In this study, we used existing sampling data from the low flow period of 2021 to predict species richness using environmental variables such as elevation, mean annual precipitation, and slope or confinement of the river.

Species richness almost uniformly increased across the study area as elevation decreased from the Andes toward the Pacific Ocean. While this relationship is not novel and aligns with previous research (Schlosser 1987; Thorp et al. 2008; Pease et al. 2012; Allan et al. 2021), the use of MSOM to predict species richness in this region of Chile explicitly highlights local trends that can aid in on‐the‐ground management. For example, these maps show predicted species richness peaks in the Valdivia River drainage basin. Sampling effort in the Valdivia River drainage basin has been concentrated in the San Pedro River and in the Cruces River, where researchers found high levels of species richness and endemism (Habit and Victoriano 2012; Colin et al. 2022, 2023). Yet, the broader river network remains under‐sampled overall, making our study's model findings novel and underscoring the need for increased conservation attention in the Valdivia River drainage basin specifically.

The Puelo drainage basin is also predicted to contain elevated species richness rates even though it has a lower pool of potential species (gamma diversity) versus other drainage basins in our study area due to its geographic isolation. This result could be influenced by the relationship between mean annual precipitation and species richness in the model. Although species richness did not have a statistically significant relationship with mean annual precipitation or valley slope, including these covariates still resulted in the best model fit, and several individual species did demonstrate significant relationships. Several studies showed that, in addition to longitudinal changes in fish assemblage driven by factors like elevation (Vannote et al. 1980), hydrogeomorphology (valley slope) and climatic factors (precipitation) also drive changes to fish assemblages (Thorp et al. 2006; Maasri et al. 2021; Yarnell and Thoms 2022; Pyron et al. 2025). Increased understanding of these environmental filters can help guide better fitting models and provide a baseline of current species assemblages, allowing for future comparison under changing climatic conditions (Soranno et al. 2010).

While our results show the model covariates impact native species richness, it is also important to consider the impacts of disturbance and nonnative species on richness patterns, especially in the context of metacommunity dynamics (Gaston 2000; Allan et al. 2021). Metacommunity is defined as a group of interacting communities connected through the dispersal of different species (Wilson 1999; Stroud et al. 2015). Metacommunity dynamics, specifically dispersal rates and ability, often have the most influence on community assembly in newly formed habitat patches (after disturbance), while older patches were most influenced by habitat characteristics. Disturbance, especially frequent intense disturbance like the flood and droughts that are becoming more extreme with climate change, can reduce the diversity of native species that can survive those conditions and dramatically alter species assemblage composition (Angermeier and Schlosser 1989). These disturbances could impact species richness in central‐southern Chile (30°–48° S) where climate projections show a drying trend with decreased rainfall and total water availability, increasing the likelihood of extreme drought (Garreaud et al. 2017; Boisier et al. 2018; Villablanca et al. 2022). Longer‐term monitoring and multi‐year modeling of the system may shed light on how freshwater assembly and disassembly dynamics may unfold across central Chile.

### 4.2 Individual Species Predicted Occupancy Maps

#### 4.2.1 Nonnative Species

Nonnative species in Chile have been introduced for over a century and pose a threat to the integrity of Chile's native fish assemblages (Figure 3). *Gambusia* species (mosquitofish) were introduced to control mosquitos in the early 1900s and impacted native fishes in warmer streams throughout Chile, including the *Galaxiidae* species (Welcomme 1988; Habit et al. 2012). Mosquito fish are particularly well adapted to rapidly colonize new habitats given their biology and opportunistic life history (Winemiller and Rose 1992). Studies show mosquitofish in Chile, along with several other exotic species possessing functional traits such as small laterally compressed bodies and generalist diets, have contributed to the homogenization of native fish assemblages and reduced functional diversity (Rojas et al. 2020). Reductions in functional diversity can undermine the resilience and functioning of aquatic ecosystems, making them more vulnerable to environmental changes, less productive, and less capable of providing essential services (Olden et al. 2010; Villéger et al. 2017). *Cyprinus carpio* (carp) were found in lower abundance in our predicted occupancy maps; however, they have also been grouped with salmonids and mosquitofish in causing declines of native freshwater species in the United States through predation and competition and are capable of spreading infection to native species (Rahel 2000; Habit et al. 2024). Our findings align with previous studies that show carp and mosquito fish occur more frequently in low elevations where native species richness is higher and are often found in degraded rivers (Figure 3) (Duarte et al. 1971; Habit et al. 2010, 2012, 2024). Combining the results of our species richness maps with carp and mosquito distribution could aid managers in prioritizing the conservation of highly diverse rivers that have not yet been invaded, as well as targeting the removal of these nonnative species.

Salmonids have been widely introduced, initially by government initiatives in the early 1900s for recreational fishing and aquaculture (Basulto 2003). The predicted occupancy maps for these species are consistent with their biology. Whereas *S. trutta* (Brown trout) is primarily found in the coldest southern regions, *O. mykiss* (Rainbow trout) with their higher thermal tolerance, have successfully spread throughout the majority of high/mid elevation streams in the study region. Both species are most abundant in high elevation streams characterized by high dissolved oxygen and low water temperatures. Unfortunately, these salmonids compete with native coldwater fishes for resources and can exhibit massive predatory pressure (Ruiz and Berra 1994; Macchi et al. 1999; Habit et al. 2010). There is also evidence that the introduction of nonnative species can play a role in the taxonomic homogenization of formerly distinct regional fish communities, resulting in a loss of native biodiversity overall (Vargas et al. 2015).

While there have been negative impacts of salmonids on native fish populations, they also support a thriving multimillion dollar recreational fishing industry in Chile, which has resulted in increased calls for ecosystem conservation (Soto et al. 2006; Arismendi and Nahuelhual 2007; Habit et al. 2012). Balancing ecological and economic considerations is crucial for both native species and salmonids. Soto et al. (2006) proposed increasing salmonid fishing pressure in the central valley streams to decrease the impacts of these species on native fish while still maintaining the recreational fishery. This idea could also be expanded to include increased salmonid fishing pressure in high elevation streams where catch and release is currently mandated. Results from this study may be useful in guiding the management and protection of areas yet to be invaded outside of our study area or areas to target higher fishing pressure.

#### 4.2.2 Data Deficient Species

MSOMs are especially valuable for species with limited data, such as the IUCN data deficient species *G. australis* and *T. areolatus* , as these models can provide insights into distribution and potential conservation needs (Reis and Lima 2007; Baker and Bice 2022) (Figure 4). *Geotria australis* , also known as the pouched lamprey, occurs throughout Australia, New Zealand, and Chile (Baker and Bice 2022). A total of > 25% of lamprey species worldwide are at risk of extinction and are therefore in need of increased protection and management, which can be informed by species distribution data (Maitland et al. 2015; Lucas et al. 2021). However, given the pouched lamprey's preference for cryptic habitats and sparse monitoring, data on its distribution and population size are limited (Miller et al. 2021). Studies from other countries indicate population decline, especially in areas impacted by dams, as they impede anadromous migration and migratory cues specific to the natural flow regime (Liermann et al. 2012). It is also listed as vulnerable by the Ministry of Environment in Chile, and Díaz et al. (2021) found this species absent in highly fragmented Chilean river basins. Given this species' high occurrence rates in the southernmost part of the study area, conservation in this area could be prioritized. The ability of MSOMs to estimate occupancy for species with sparse data offers a significant advantage, allowing for more informed decision‐making even when traditional data sources are lacking.

*Trichomycterus areolatus* is an endemic freshwater Chilean species of pencil catfish found in high‐gradient riffles and rapid habitats (Schulze et al. 2016). Schulze et al. (2016) provide an excellent example of how this catfish could be used as an indicator species of ecosystem health given its unique adaptations to Chile's high‐gradient watersheds and point to other relevant studies that have attempted to do this generally (Arciszewski and Munkittrick 2015) and as indicators of paper mill contamination with another IUCN‐listed species *P. gillissi* (Chiang et al. 2011). While an older report indicated this species may be declining across its range (Campos et al. 1998), the predicted occupancy map indicates it may be quite widespread with somewhat high abundance, perhaps due to an ability to rapidly colonize disturbed areas (Bañales‐Seguel et al. 2024). These results could improve targeted monitoring to ground‐truth the model and assess the status of the population and if it can be used as an effective indicator species.

#### 4.2.3 IUCN Vulnerable and Endangered Species

*Percilia irwini* and *P. gillissi* are both IUCN endangered species endemic to Chile. Both species are impacted by habitat degradation from the construction of hydropower dams and *P. irwini* has experienced sharp declines in its abundance and genetic diversity (Habit et al. 2007; Habit et al. 2019; Valenzuela‐Aguayo et al. 2020). This decline is especially worrisome given the numerous planned dams in the Biobío River drainage basin and the additive impacts of climate change in this vulnerable Mediterranean climate region (Díaz et al. 2019; Araya‐Osses et al. 2020). Additionally, both species are impacted by predation from nonnative salmonids (Vila and Habit 2015; Habit et al. 2019). While both species are classified at the national level as “endangered” by the Ministry of Environment (mma.gob.cl), no formal conservation actions or management and monitoring plans are in place outside of protected zones (national parks and reserves). Our results indicate that *P. gillissi* higher rates of predicted occupancy at lower elevations along the coast could help guide conservation planning as many of the current and planned dams are found at these elevations.

The predicted fish occurrence probabilities are based in part on the relationship of fish occurrence with the model covariates (elevation, mean annual precipitation, and confinement). At the Biobío River drainage basin scale, covariates exhibit less variation compared to the broader 11‐basin analysis, resulting in a weaker predictive relationship and reduced model accuracy for the smaller, more homogeneous study area. This may be attributed to the smaller sample size and limited range of explanatory variables within the Biobío. Attempts to refine the model by adjusting covariates did not resolve the observed disparities between predicted occurrence and on‐the‐ground sampling. Response plots further confirmed weaker covariate relationships in the Biobío model compared to the larger scale model. The results highlight the importance of researchers and managers exercising caution if relying solely on these predicted maps to assess species predicted occurrence. While these maps can be a helpful guide for activities such as identifying additional sampling locations, they should not be used as definitive species presence guides and should be used in combination with other more local, fine‐scale resources.

### 4.3 Dam Impacts on Native Species and Conservation Implications

Dams are a quintessential “wicked problem,” offering a source of renewable energy while simultaneously disrupting riverine ecosystems (Lönngren and van Poeck 2021). Several recent studies have explored the effects of hydroelectric dams on Chilean rivers specifically, including their freshwater fish populations. These studies have emphasized the need for more research on species' basic ecology to inform strategic dam planning and impact assessments (Habit et al. 2007, 2019, 2024; Laborde et al. 2020; Díaz et al. 2021, 2023; Villablanca et al. 2022). The species richness and individual occupancy modeling results from MSOMs may aid in illuminating the potential of dam impacts in data‐limited systems and highlight areas of conservation concern.

Habit et al. (2024) provide a comprehensive overview of the impacts of anthropogenic pressures on native freshwater fish in Chile and suggest that the loss of longitudinal connectivity and natural flow regime due to dams may be most severe in the central and southern zones basins (Maipo, Rapel, Mataquito, Maule, Itata, and Biobío). Similar impacts of dams are widely known from other parts of the world (Dynesius and Nilsson 1994; Richter et al. 2010; Xu et al. 2013). Our modeling results explicitly reveal how dams planned in lower elevation rivers will impact species richness and thus should be viewed as priority conservation areas (Habit et al. 2024). Additionally, some dams planned in higher elevation reaches of the rivers may have severe impacts on river habitat and the fishes they support downstream. For example, changes to key habitat variables including substrate, temperature, flow, organic matter, and habitat availability are expected (Copaja et al. 2016; Moraga et al. 2022; Díaz et al. 2023). Hydroelectric plants in Chile are also known to lead to flow alterations (Habit et al. 2007) and hydropeaking impacts (García et al. 2011; Elgueta et al. 2021) that can reduce habitat availability and survival of native fishes.

The type and location of hydropower facilities or dams can also impact fish communities in a variety of different ways. There is a well‐established evidence base that large dams can impede fish passage for migratory (Díaz et al. 2021; Miller et al. 2021) and nonmigratory species (Oyanedel et al. 2018; Ramírez‐Álvarez et al. 2022) and ultimately erode levels of alpha and beta diversity (Díaz et al. 2023). Recently, a study analyzing the impacts of a newly constructed low‐head diversion in central Chile found that the dam greatly reduced species richness in one of the affected rivers that was already highly disturbed by other dams, while impacts to the nearby relatively undisturbed river were more minimal (Benstead et al. 1999; Bratrich et al. 2004; Habit et al. 2007). This study also showed that while these low‐head dams were unlikely to impact large‐bodied fish migration, they may impede native smaller‐bodied fish from upstream movement (Habit et al. 2007).

Given the varied impacts dams can have on rivers and the fish communities they support, it is important to ensure managers have the information they need to tailor mitigation strategies. One strategy to maintain higher diversity is to prioritize conservation of longer stream networks, unimpaired by barriers, as they contain a greater variety of habitat types that ultimately support a more diverse assemblage of species and offer refugia habitat during disturbance (Pringle 2001; Arantes et al. 2019). If barriers exist, previous studies suggest implementing fishways and other mitigation technologies (screens and bypasses to prevent fish injury and death) tailored to the specific fish of the region to allow for passage (Branco et al. 2017; Romão et al. 2018; Laborde et al. 2020). Laborde et al. (2020) found that species belonging to *Trichomycterus*, *Diplomystes*, and *Percilia* genera were particularly at risk in central Chile due to hydropower barriers and proposed designing fish passage structures to support their unique biological requirements (Laborde et al. 2020). The results of this study can aid managers in identifying critical habitats and migration corridors, which in turn guide dam planning, removal, and implementation of fish passage structures. Species occupancy maps could also guide future monitoring and assessment of native species before and after dams are built to assess their impact on native and nonnative species. Finally, predicted species richness maps may help managers grapple with trade‐offs that acknowledge the reality of hydropower on the landscape but also attempt to minimize biodiversity loss.

## 5 Conclusion

Chilean rivers support a unique and highly endemic assemblage of freshwater fishes, many of which are vulnerable to disturbances such as dam construction and climate change. Our study demonstrates the utility of multispecies occupancy models in predicting species richness and occupancy patterns across diverse hydro‐geomorphological settings when comprehensive monitoring data are lacking. By providing a framework that accounts for both species‐specific responses and community‐level dynamics, MSOMs can guide large‐scale conservation planning, help identify biodiversity hotspots, and assess aspects of ecosystem resilience. Moreover, MSOMs can aid in anticipating species distribution shifts under future climate and land use scenarios, providing tools for proactive management to mitigate biodiversity loss before it occurs. As Chile continues to strike a balance between societal and environmental needs, MSOMs can play a crucial role in enhancing the resilience of Chilean freshwater ecosystems to future disturbances and ensuring native species live on.

Beyond their application in Chile, MSOMs offer a powerful tool for fisheries conservation globally. By simultaneously modeling multiple species, these models infer both species‐specific and community‐level responses to environmental conditions while accounting for imperfect detection, species interactions, and spatial autocorrelation. This makes MSOMs particularly valuable in areas with limited monitoring data, such as infrequent surveys or sparse fish life history information. By addressing species interactions and integrating detection probabilities, MSOMs provide deeper insights into habitat use, community dynamics, and the impacts of environmental gradients. These attributes make MSOMs highly relevant for informing fisheries management and conservation efforts, offering a framework that supports both local and global strategies to conserve aquatic biodiversity.

## Author Contributions

**Erin E. Tracy:** conceptualization (lead), data curation (lead), formal analysis (lead), funding acquisition (lead), investigation (lead), methodology (lead), visualization (lead), writing – original draft (lead), writing – review and editing (equal). **Evelyn Habit:** conceptualization (supporting), data curation (equal), formal analysis (supporting), investigation (supporting), writing – review and editing (supporting). **Konrad Górski:** conceptualization (supporting), data curation (supporting), formal analysis (supporting), investigation (supporting), writing – review and editing (supporting). **Nann A. Fangue:** conceptualization (supporting), formal analysis (supporting), investigation (supporting), methodology (supporting), supervision (equal), writing – review and editing (supporting). **Andrew L. Rypel:** conceptualization (supporting), formal analysis (supporting), investigation (supporting), methodology (supporting), supervision (equal), writing – review and editing (supporting).

## Conflicts of Interest

The authors declare no conflicts of interest.

## Supporting information


**Table S1** Occurrence values from MSOM model. The ratio of valley width to valley floor width is labeled as confinement and mean annual precipitation is labeled ppt.
**Table S2** Detection values from the MSOM.

## Data Availability

The data that support the findings of this study are openly available in Dryad, https://doi.org/10.5061/dryad.7d7wm385c.
