# Supplementary material for: Assessing Patterns and Risk to Chilean Freshwater Fish Distributions Using Multi‐Species Occupancy Models
Source: Ecol Evol. 2025 Jul 15;15(7):e71719. doi: 10.1002/ece3.71719 (PMC12263197; doi:10.1002/ece3.71719)
Supplement: Supplementary file 1 — Table S1 Occurrence values from MSOM model. The ratio of valley width to valley floor width is labeled as confinement and mean annual precipitation is labeled ppt. Table S2 Detection values from the MSOM. [file ECE3-15-e71719-s001.docx]

**Supplemental Table 1.** Occurrence values from MSOM model. The ratio of valley width to valley floor width is labeled as confinement and mean annual precipitation is labeled ppt.

| **Occurrence logit scale variable** | **Mean** | **SD** | **2.5%** | **50%** | **97.5%** |
| --- | --- | --- | --- | --- | --- |
| occurrence mean intercept | -2.23 | 0.72 | -3.64 | -2.24 | -0.78 |
| occurrence mean elevation | -1.44 | 0.45 | -2.38 | -1.42 | -0.59 |
| occurrence mean ppt | -0.10 | 0.54 | -1.17 | -0.09 | 0.95 |
| occurrence mean confinement | 0.04 | 0.22 | -0.39 | 0.04 | 0.47 |
| occurrence variance intercept | 12.47 | 5.48 | 5.37 | 11.31 | 26.23 |
| occurrence variance elevation | 3.13 | 1.77 | 0.98 | 2.72 | 7.65 |
| occurrence variance ppt | 6.08 | 3.33 | 2.17 | 5.32 | 14.42 |
| occurrence variance confinement | 0.20 | 0.21 | 0.03 | 0.14 | 0.71 |
| elevation *Trichomycterus areolatus* | 1.03 | 0.77 | -0.40 | 1.00 | 2.67 |
| elevation *Aplochiton taeniatus* | -1.72 | 1.38 | -4.76 | -1.62 | 0.66 |
| elevation *Geotria australis* | -2.33 | 0.88 | -4.24 | -2.27 | -0.80 |
| elevation *Gambusia holbrooki* | -2.80 | 1.05 | -5.12 | -2.70 | -1.02 |
| elevation *Percichthys trucha* | -2.13 | 0.88 | -4.04 | -2.06 | -0.61 |
| elevation *Cyprinus carpio* | -1.84 | 1.34 | -4.71 | -1.76 | 0.65 |
| elevation *Percilia gillissi* | -2.00 | 0.87 | -3.89 | -1.94 | -0.49 |
| elevation *Cheirodon galusdae* | 0.10 | 1.19 | -1.93 | -0.02 | 2.81 |
| elevation *Basilichthys microlepidotus* | -2.03 | 0.99 | -4.26 | -1.91 | -0.40 |
| elevation *Percilia irwini* | -0.37 | 0.75 | -1.90 | -0.35 | 1.05 |
| elevation *Oncorhynchus mykiss* | 1.41 | 0.72 | 0.17 | 1.34 | 3.01 |
| elevation *Galaxias maculatus* | -3.16 | 0.95 | -5.30 | -3.06 | -1.57 |
| elevation *Salmo trutta* | -1.03 | 0.98 | -3.07 | -0.98 | 0.80 |
| elevation *Bullockia maldonadoi* | -2.10 | 0.88 | -4.00 | -2.04 | -0.54 |
| elevation *Diplomystes nahuelbutaensis* | 0.67 | 1.10 | -1.19 | 0.55 | 3.31 |
| elevation *Diplomystes incognitus* | -0.03 | 1.22 | -2.42 | -0.04 | 2.57 |
| elevation *Cnesterodon decemmaculatus* | -2.58 | 1.19 | -5.16 | -2.49 | -0.49 |
| elevation *Australoheros facetus* | -3.06 | 1.44 | -6.22 | -2.94 | -0.57 |
| elevation *Cheirodon australe* | -2.69 | 1.33 | -5.70 | -2.55 | -0.46 |
| elevation *Aplochiton marinus* | -2.01 | 1.36 | -5.00 | -1.91 | 0.36 |
| elevation *Diplomystes camposensis* | -2.46 | 1.29 | -5.26 | -2.37 | -0.17 |
| elevation *Cheirodon kiliani* | -1.76 | 1.38 | -4.75 | -1.66 | 0.64 |
| elevation *Hatcheria macraei* | -1.77 | 1.42 | -4.85 | -1.69 | 0.76 |
| elevation *Brachygalaxias bullock* | -1.84 | 1.49 | -5.01 | -1.75 | 0.87 |
| elevation *Galaxias platei* | -1.22 | 1.21 | -3.76 | -1.17 | 1.00 |
| ppt *Trichomycterus areolatus* | -3.00 | 1.08 | -5.36 | -2.93 | -1.09 |
| ppt *Aplochiton taeniatus* | 0.32 | 1.49 | -2.31 | 0.24 | 3.62 |
| ppt  *Geotria australis* | 1.71 | 0.86 | 0.51 | 1.56 | 4.04 |
| ppt *Gambusia holbrooki* | -4.16 | 1.48 | -7.73 | -3.92 | -1.99 |
| ppt *Percichthys trucha* | -0.79 | 0.92 | -2.62 | -0.79 | 1.16 |
| ppt *Cyprinus carpio* | -2.48 | 1.54 | -6.23 | -2.22 | -0.19 |
| ppt *Percilia gillissi* | 0.26 | 0.86 | -1.38 | 0.22 | 2.05 |
| ppt *Cheirodon galusdae* | -1.40 | 1.48 | -5.10 | -1.17 | 0.89 |
| ppt *Basilichthys microlepidotus* | -1.48 | 1.00 | -3.65 | -1.41 | 0.29 |
| ppt *Percilia irwini* | -0.02 | 1.10 | -2.15 | -0.03 | 2.20 |
| ppt *Oncorhynchus mykiss* | 2.98 | 1.08 | 1.23 | 2.86 | 5.44 |
| ppt *Galaxias maculatus* | 1.96 | 1.13 | 0.41 | 1.80 | 4.74 |
| ppt *Salmo trutta* | 1.41 | 0.99 | -0.37 | 1.35 | 3.54 |
| ppt *Bullockia maldonadoi* | -0.04 | 0.89 | -1.75 | -0.05 | 1.79 |
| ppt *Diplomystes nahuelbutaensis* | 0.23 | 1.18 | -2.07 | 0.20 | 2.65 |
| ppt *Diplomystes incognitus* | -2.01 | 1.58 | -5.58 | -1.84 | 0.58 |
| ppt *Cnesterodon decemmaculatus* | -3.08 | 1.37 | -6.14 | -2.94 | -0.82 |
| ppt *Australoheros facetus* | -1.85 | 1.12 | -4.38 | -1.74 | 0.04 |
| ppt *Cheirodon australe* | 1.33 | 1.13 | -0.21 | 1.09 | 4.34 |
| ppt *Aplochiton marinus* | -1.61 | 1.35 | -4.77 | -1.44 | 0.54 |
| ppt *Diplomystes camposensis* | 2.81 | 1.13 | 1.10 | 2.65 | 5.47 |
| ppt *Cheirodon kiliani* | 0.17 | 1.53 | -2.69 | 0.12 | 3.55 |
| ppt *Hatcheria macraei* | 1.21 | 1.21 | -0.87 | 1.11 | 3.84 |
| ppt *Brachygalaxias bullocki* | 1.31 | 1.54 | -1.26 | 1.15 | 4.81 |
| ppt *Galaxias platei* | 3.57 | 1.26 | 1.56 | 3.41 | 6.50 |
| confinement *Trichomycterus areolatus* | -0.03 | 0.34 | -0.71 | -0.03 | 0.64 |
| confinement *Aplochiton taeniatus* | 0.04 | 0.46 | -0.89 | 0.04 | 0.94 |
| confinement *Geotria australis* | 0.08 | 0.38 | -0.68 | 0.08 | 0.84 |
| confinement *Gambusia holbrooki* | -0.07 | 0.48 | -1.11 | -0.05 | 0.82 |
| confinement *Percichthys trucha* | -0.08 | 0.38 | -0.86 | -0.07 | 0.65 |
| confinement *Cyprinus carpio* | -0.02 | 0.47 | -1.01 | -0.01 | 0.88 |
| confinement *Percilia gillissi* | 0.19 | 0.44 | -0.62 | 0.17 | 1.13 |
| confinement *Cheirodon galusdae* | -0.18 | 0.50 | -1.30 | -0.14 | 0.71 |
| confinement *Basilichthys microlepidotus* | 0.10 | 0.44 | -0.76 | 0.09 | 0.98 |
| confinement *Percilia irwini* | -0.09 | 0.42 | -0.97 | -0.07 | 0.72 |
| confinement *Oncorhynchus mykiss* | 0.22 | 0.45 | -0.60 | 0.19 | 1.20 |
| confinement *Galaxias maculatus* | 0.06 | 0.35 | -0.63 | 0.06 | 0.74 |
| confinement *Salmo trutta* | 0.01 | 0.38 | -0.77 | 0.01 | 0.77 |
| confinement *Bullockia maldonadoi* | 0.19 | 0.38 | -0.53 | 0.18 | 0.97 |
| confinement *Diplomystes nahuelbutaensis* | 0.18 | 0.44 | -0.65 | 0.17 | 1.10 |
| confinement *Diplomystes incognitus* | 0.00 | 0.48 | -1.00 | 0.01 | 0.92 |
| confinement *Cnesterodon decemmaculatus* | 0.12 | 0.47 | -0.80 | 0.11 | 1.11 |
| confinement *Australoheros facetus* | 0.04 | 0.46 | -0.92 | 0.04 | 0.95 |
| confinement *Cheirodon australe* | 0.13 | 0.42 | -0.66 | 0.12 | 1.01 |
| confinement *Aplochiton marinus* | -0.01 | 0.47 | -1.00 | 0.01 | 0.90 |
| confinement *Diplomystes camposensis* | 0.25 | 0.44 | -0.56 | 0.22 | 1.19 |
| confinement *Cheirodon kiliani* | 0.02 | 0.46 | -0.94 | 0.03 | 0.91 |
| confinement *Hatcheria macraei* | -0.20 | 0.49 | -1.30 | -0.15 | 0.64 |
| confinement *Brachygalaxias bullocki* | -0.15 | 0.50 | -1.26 | -0.11 | 0.74 |
| confinement *Galaxias platei* | 0.13 | 0.42 | -0.68 | 0.12 | 1.00 |

**Supplemental Table 2.** Detection values from the MSOM.

| **Detection logit scale variable** | **Mean** | **SD** | **2.5%** | **50%** | **97.5%** |
| --- | --- | --- | --- | --- | --- |
| ***community*** |  |  |  |  |  |
| detection mean | 1.00 | 0.41 | 0.12 | 1.02 | 1.76 |
| detection variance | 2.48 | 1.56 | 0.70 | 2.07 | 6.52 |
| ***species*** |  |  |  |  |  |
| *Trichomycterus areolatus* | 2.15 | 0.38 | 1.47 | 2.13 | 2.94 |
| *Aplochiton taeniatus* | 0.40 | 2.05 | -3.84 | 0.70 | 3.95 |
| *Geotria australis* | 2.02 | 0.71 | 0.73 | 1.99 | 3.52 |
| *Gambusia holbrooki* | 1.50 | 0.63 | 0.42 | 1.44 | 2.89 |
| *Percichthys trucha* | 0.31 | 0.35 | -0.34 | 0.29 | 1.03 |
| *Cyprinus carpio* | -0.19 | 0.99 | -1.88 | -0.30 | 2.02 |
| *Percilia gillissi* | 2.94 | 0.77 | 1.66 | 2.87 | 4.66 |
| *Cheirodon galusdae* | -0.39 | 0.38 | -1.11 | -0.39 | 0.37 |
| *Basilichthys microlepidotus* | 0.04 | 0.35 | -0.60 | 0.02 | 0.77 |
| *Percilia irwini* | 3.62 | 1.02 | 2.06 | 3.47 | 6.06 |
| *Oncorhynchus mykiss* | 2.24 | 0.46 | 1.41 | 2.21 | 3.22 |
| *Galaxias maculatus* | 1.98 | 0.57 | 0.94 | 1.95 | 3.18 |
| *Salmo trutta* | 0.74 | 0.37 | 0.05 | 0.73 | 1.51 |
| *Bullockia maldonadoi* | 0.62 | 0.63 | -0.51 | 0.58 | 1.96 |
| *Diplomystes nahuelbutaensis* | 0.26 | 0.65 | -0.86 | 0.21 | 1.68 |
| *Diplomystes incognitus* | 0.27 | 1.82 | -3.35 | 0.37 | 3.75 |
| *Cnesterodon decemmaculatus* | 1.87 | 1.26 | -0.27 | 1.75 | 4.71 |
| *Australoheros facetus* | 1.87 | 1.28 | -0.34 | 1.75 | 4.73 |
| *Cheirodon australe* | 0.34 | 0.66 | -0.80 | 0.29 | 1.69 |
| *Aplochiton marinus* | 0.66 | 1.63 | -2.41 | 0.67 | 3.93 |
| *Diplomystes camposensis* | 1.44 | 0.55 | 0.42 | 1.42 | 2.59 |
| *Cheirodon kiliani* | 0.39 | 2.05 | -3.88 | 0.70 | 3.90 |
| *Hatcheria macraei* | 0.48 | 1.26 | -2.38 | 0.56 | 2.80 |
| *Brachygalaxias bullocki* | -0.94 | 1.46 | -3.94 | -0.79 | 1.55 |
| *Galaxias platei* | 1.26 | 0.51 | 0.32 | 1.24 | 2.30 |
